# Effectiveness and mechanisms of Intensive Short-Term Dynamic Psychotherapy for treatment-resistant depression: a reanalysis of a randomized controlled trial

**DOI:** 10.3389/fpsyt.2026.1815946

**Published:** 2026-04-22

**Authors:** Robert Johansson, Peter Lilliengren

**Affiliations:** Department of Psychology, Stockholm University, Stockholm, Sweden

**Keywords:** ISTDP, mediation analysis, process variables, psychodynamic therapy, psychotherapy, randomized controlled trial, reanalysis, treatment-resistant depression

## Abstract

**Introduction:**

Intensive Short-Term Dynamic Psychotherapy has shown promising effects for treatment-resistant depression, but it remains unclear whether its hypothesized mechanisms — reducing emotional repression, negative affect, and psychological distress — actually mediate treatment outcomes.

**Methods:**

We reanalyzed publicly available data from a randomized controlled trial (*N* = 86) comparing 20 sessions of Intensive Short-Term Dynamic Psychotherapy to waitlist control for treatment-resistant depression. Depression and process measures were assessed at baseline, post-treatment, and 3-month follow-up. Linear mixed-effects models analyzed trajectories; bootstrap mediation and cross-lagged panel analyses provided an exploratory examination of proposed process variables.

**Results:**

Treatment produced large effects on depression at post-treatment (Cohen’s *d* = 1.68) that continued to increase through 3-month follow-up (*d* = 2.50, 95% CI [1.88, 3.11]). All proposed process measures also showed very large effects (*d* = 1.96–2.95). However, neither emotional repression nor negative affect significantly mediated depression improvement. Distress showed apparent mediation, but a sensitivity analysis removing the overlapping depression subscale eliminated this effect entirely, confirming it reflected construct overlap rather than a genuine indirect effect. Cross-lagged analyses revealed no temporal precedence for any process measure, indicating concurrent rather than sequential change.

**Discussion:**

These findings confirm that this psychotherapy produces large, durable effects on treatment-resistant depression. However, the theorized sequential process — whereby reducing defensive functioning leads to improved affect regulation, which in turn alleviates depression — was not supported in the present data. Instead, the treatment appears to produce broad, simultaneous therapeutic change across multiple psychological domains. These exploratory findings suggest that understanding how psychotherapy works may require finer temporal measurement and observational methods that capture in-session processes.

## 1 Introduction

Depression is one of the most prevalent and debilitating mental health conditions worldwide, affecting an estimated 280 million individuals and representing a leading cause of disability (1). While evidence-based treatments such as antidepressant medication and psychotherapy produce meaningful improvements for many patients, a substantial proportion—approximately 30% to 40%—do not achieve remission with first line interventions (2, 3). This phenomenon, termed treatment-resistant depression (TRD), is characterized by inadequate response to at least one adequate trial of antidepressant treatment and poses one of the most formidable challenges in contemporary mental health care (4). The burden of TRD is considerable, not only for affected individuals who experience prolonged suffering and functional impairment, but also for healthcare systems facing increased service utilization and costs (5). Given the cumulative failure rates that increase with sequential treatment trials (2), there is an urgent need for effective alternative interventions that can offer relief when conventional approaches prove insufficient.

Psychotherapy represents a promising avenue for addressing TRD, with emerging evidence suggesting that structured psychological interventions can produce clinically meaningful improvements even in patients who have not responded to pharmacotherapy (6, 7). Among psychotherapeutic approaches, Intensive Short-Term Dynamic Psychotherapy (ISTDP) has garnered increasing empirical support for the treatment of depression, including treatment-resistant presentations (8, 9). ISTDP is a form of brief psychodynamic therapy developed by Habib Davanloo (10) that aims to rapidly access and resolve unconscious emotional conflicts through active techniques designed to overcome psychological defenses. Unlike traditional psychodynamic approaches that may extend over years, ISTDP is delivered in a time-limited format, typically ranging from 10 to 40 weekly individual sessions (11). The approach is characterized by systematic attention to the therapeutic relationship, active challenge of maladaptive defenses, and facilitation of direct emotional experiencing of previously avoided feelings.

The theoretical framework underlying ISTDP proposes that depression, like other forms of psychopathology, arises fundamentally from unprocessed attachment trauma and the defensive avoidance of painful emotions associated with early relational experiences (11, 12). According to this model, attachment related injuries—such as loss, neglect, criticism, or abuse—evoke powerful reactive emotions in the developing child, particularly anger directed toward attachment figures who have been experienced as frustrating, rejecting, or threatening (13). However, because the child’s psychological and physical survival depends on maintaining proximity to these same attachment figures, the direct expression of anger is experienced as dangerous and is therefore defended against through various psychological mechanisms. In the specific case of depression, the theory posits that the child may internalize the critical, judgmental, or threatening attributes of the attachment figure through identification, turning anger inward and developing a pattern of self-attack that mirrors the original external threat (12, 13). This internalization gives rise to the regressive defenses characterizing depression—including self-criticism, self-destructiveness, resignation, and agonizing—which serve to distance the individual from the conflicting painful feelings associated with the frustrating other (12). Unconscious rage, guilt about this rage, and grief over what was lost or never received are believed to lie at the root of depressive symptomatology, with the chronic avoidance of these complex emotions driving the depression forward over time (11, 13).

The ISTDP treatment approach follows directly from this etiological model. The therapist actively works to help patients relinquish their defensive strategies and directly experience the previously avoided emotions associated with past attachment trauma, thereby revealing the internal dynamics that maintain the depression (11, 14). Treatment is always adapted to the individual patient’s level of anxiety tolerance and defensive organization, with specific attention in depressed patients to their characteristic difficulties with intimacy and closeness, vulnerability to loss and separation, and resistance to the therapeutic relationship itself (11, 13). The technique involves addressing the patient’s stated desire to feel better while simultaneously challenging the patient’s defenses against closeness and emotional experiencing. Through this process, the aim is to access and make conscious the unconscious complex emotions—particularly anger, guilt, and grief—associated with past attachment trauma, thereby dismantling the defensive structures that generate and perpetuate depressive symptoms.

Empirical research on ISTDP has accumulated steadily over the past two decades, with meta-analytic evidence indicating moderate to large effects across a range of psychiatric conditions (8, 15, 16). For depression specifically, multiple randomized controlled trials have demonstrated that ISTDP produces clinically significant improvements compared to control conditions receiving usual care or minimal intervention (9, 17–19). When compared to waitlist control conditions, ISTDP has been shown to produce large effect sizes for both depressive symptoms and associated interpersonal difficulties, with effects maintained at follow-up assessments ranging from 3 to 12 months (20, 21). Importantly, these beneficial effects have been observed specifically in treatment-resistant populations. Town and colleagues (9, 22) conducted a randomized controlled trial of ISTDP versus treatment as usual in patients with treatment-resistant depression, finding large effects on depressive symptoms that were sustained through an 18-month follow-up period, along with significant reductions in healthcare costs.

Recent large-scale studies have further strengthened the evidence base for ISTDP in depression. Following a pilot single-subject study showing promising initial results (23), Heshmati et al. (24) conducted a randomized controlled trial with 86 Iranian adults diagnosed with treatment-resistant depression, comparing 20 sessions of ISTDP delivered over 10 weeks to a waitlist control condition. The study found very large effects on depressive symptoms (Cohen’s *d* = 1.73 at post-treatment, increasing to *d* = 2.67 at 3-month follow-up), along with substantial improvements in emotional repression and negative affect. Similarly, Johansson et al. (13) examined ISTDP effectiveness in a naturalistic sample of 195 patients with depression treated at a specialty clinic in Halifax, Canada, finding large within-group effects on both depression (Cohen’s *d* = 1.02) and interpersonal problems (Cohen’s *d* = 1.17). Across these studies, the consistency and magnitude of observed effects suggest that ISTDP represents a robust and effective intervention for depression, including presentations that have proven resistant to conventional treatments.

Despite this accumulating evidence that ISTDP works for depression, a critical question remains largely unanswered: *How* does ISTDP work? Understanding the processes through which therapeutic interventions produce their effects is essential not only for refining theoretical models, but also for optimizing treatment delivery, improving training programs, and identifying which patients are most likely to benefit from specific therapeutic approaches (25, 26). The theoretical model articulated above proposes a specific sequential process: ISTDP techniques help patients reduce defensive avoidance of emotions (i.e., reduce emotional repression), which in turn allows for the experience and processing of previously avoided feelings (reducing negative affect and distress), which ultimately produces relief from depressive symptoms. This theoretical pathway implies that changes in emotional repression and affect should not merely co-occur with depression improvement, but should actually *mediate* that improvement—that is, serve as processes through which treatment exerts its effects.

Some empirical evidence supports the proposed mediating role of emotional experiencing in ISTDP. Johansson et al. (27) and Town et al. (28) found that “unlocking the unconscious”—a process characterized by the patient accessing and expressing previously repressed emotions, often accompanied by visual associations linking current feelings to past attachment figures—was associated with reduced symptom levels and fewer interpersonal problems. More recently, Johansson et al. (13) examined this process as a formal mediator in their naturalistic sample of depressed patients, finding that the occurrence of unlocking during treatment significantly mediated outcomes, with a between-group effect size of *d* = 0.60 for depression and *d* = 0.47 for interpersonal problems. Patients who experienced unlocking during treatment showed larger improvements (depression *d* = 1.32) compared to those who did not (*d* = 0.72). Similarly, Town et al. (29) investigated in-session affect experiencing as a mechanism in an RCT for treatment-resistant depression, finding that experiencing anger during sessions predicted subsequent reductions in depressive symptoms, particularly for patients with lower levels of personality pathology, and that this relationship was mediated by therapeutic alliance and insight.

However, these promising findings are tempered by important methodological limitations. Most existing studies of ISTDP process variables have relied on concurrent associations between process measures and outcomes, which cannot establish the temporal ordering necessary to infer causation (30). Even when formal mediation analyses have been conducted, they have typically examined change scores calculated across the same time period for both mediator and outcome, leaving open the question of whether process changes actually *precede* and cause symptom changes, or whether both simply change together in response to some other factor (25). The Heshmati et al. (24) study, despite demonstrating very large effects on both depression and proposed process measures (emotional repression, negative affect, overall distress), did not test whether changes in these process variables mediated the observed depression improvements, nor did it examine the temporal sequencing of changes across different domains.

The present study addresses these gaps by conducting an exploratory reanalysis of the public data from Heshmati et al. (24), applying statistical methods to examine the theoretical process variables proposed to underlie ISTDP’s effects on treatment-resistant depression. Leveraging the principles of open science that make such secondary analyses possible (31), we examined four specific research questions. First, we sought to confirm and extend the original study’s findings by examining the magnitude and durability of ISTDP’s effects on depression across all available assessment points, including the 3-month follow-up. Second, we tested whether ISTDP produces the theoretically predicted changes in proposed process measures— emotional repression, negative affect, and overall distress—with effect sizes comparable to those observed for depression itself. Third, and most critically, we conducted formal mediation analyses to test whether changes in these process variables statistically mediate the relationship between treatment assignment and depression outcomes, as the theoretical model would predict. Fourth, we employed cross-lagged panel analyses to examine temporal precedence, testing whether earlier changes in process measures predict subsequent changes in depression (consistent with a causal mediational role) or whether the temporal ordering suggests a different pattern of relationships.

We hypothesized that ISTDP would produce large effects on depression that would be maintained or increase at the 3-month follow-up assessment, consistent with the original study findings. We further hypothesized that ISTDP would produce large effects on all proposed process measures, with magnitudes comparable to effects on depression. Most importantly, we hypothesized that changes in emotional repression and negative affect would significantly mediate depression improvements, with substantial indirect effects, and that these process changes would demonstrate temporal precedence by predicting subsequent depression changes beyond what could be explained by earlier depression levels. These hypotheses follow directly from ISTDP theory. Given the moderate sample size (*N* = 86) and the exploratory nature of the process analyses, the present study should be viewed as a theory-informed examination of proposed process variables rather than a definitive test of therapeutic mechanisms. Nonetheless, findings regarding mediation and temporal precedence can inform theoretical refinement and guide future research on how ISTDP and related psychotherapies produce therapeutic change in depression.

## 2 Materials and methods

### 2.1 Study design and participants

#### 2.1.1 Overview and data source

This study presents a reanalysis of publicly available data from a randomized controlled trial examining the effects of Intensive Short-Term Dynamic Psychotherapy (ISTDP) for treatment-resistant depression (24, 32). The complete dataset, codebook, and documentation are openly accessible on the Open Science Framework (https://doi.org/10.17605/OSF.IO/75PU8). As a secondary analysis, our focus diverges from the original study by examining process-outcome relationships through advanced statistical techniques (mediation and cross-lagged analyses) that were not employed in the original publications.

#### 2.1.2 Study design

The original trial employed a two-arm parallel-group randomized controlled design with a 1:1 allocation ratio. Randomization was conducted using computer-generated random numbers, with allocations concealed in sealed, opaque envelopes opened sequentially after baseline assessment. The study was retrospectively registered on the Open Science Framework (https://osf.io/v46gy). Assessment personnel were not blinded to treatment allocation, though outcome measures were self-report questionnaires, which limits detection bias but does not eliminate potential expectancy effects.

#### 2.1.3 Recruitment and setting

Participants were recruited between April and May 2020 through referrals from psychiatrists and mental health clinics in Tabriz, Iran. Treatment was delivered between June and August 2020, with follow-up assessments completed in November 2020. The COVID-19 pandemic was ongoing during data collection, though the original report does not indicate substantial impacts on recruitment or retention.

#### 2.1.4 Participants

The study enrolled 86 Iranian adults (ages 18-60) with a current major depressive episode that had persisted for at least six weeks despite treatment with antidepressant medication. Treatment resistance was operationalized as failure to achieve adequate symptom reduction following at least one adequate trial (defined as at least 6 weeks at therapeutic dose) of an antidepressant medication from any class. All participants met DSM-IV criteria for major depressive disorder, assessed via the Mini-International Neuropsychiatric Interview-Plus (MINI-Plus).

#### 2.1.5 Participant flow

The original study screened participants referred from psychiatrists and mental health clinics; specific screening numbers were not reported in the published trial. Of those meeting eligibility criteria, 86 participants were randomized (43 to ISTDP, 43 to waitlist control). Attrition during the study was 12.8% overall: 7 participants (16.3%) withdrew from the ISTDP condition and 4 participants (9.3%) withdrew from the waitlist condition. All dropouts occurred before the post-treatment assessment, with no additional attrition between post-treatment and follow-up. At post-treatment and follow-up, data were available from 36 ISTDP participants and 39 waitlist participants. All randomized participants were included in intent-to-treat analyses using mixed-effects models with REML estimation.

#### 2.1.6 Inclusion criteria

Participants were required to: (a) be ages 18–60 years; (b) have a minimum of high school education (to ensure comprehension of self-report measures); (c) meet diagnostic criteria for current major depressive episode; (d) have treatment-resistant depression as defined above; and (e) provide informed consent.

#### 2.1.7 Exclusion criteria

Individuals were excluded for: (a) comorbid personality disorder (assessed via the MINI-Plus, which screens only for antisocial personality disorder; other personality disorders were not systematically assessed); (b) psychotic features or bipolar depression; (c) current substance dependence; (d) cognitive impairments that would interfere with psychotherapy; (e) active suicidal ideation with intent or plan requiring immediate intervention; (f) serious medical conditions requiring intensive treatment; or (g) current or recent (within 12 months) engagement in psychotherapy.

#### 2.1.8 Ethical approval

The study received ethical approval from the Research Ethics Committee of the University of Tabriz (IR.TABRIZU.REC.1400.012). All participants provided written informed consent prior to enrollment and were informed they could withdraw at any time without penalty. Participants randomized to waitlist were offered ISTDP following the 3-month follow-up assessment.

### 2.2 Measures

All measures were administered via self-report questionnaires in validated Farsi translations. Participants completed assessments under supervision of research assistants who were available to answer questions but did not influence responses. Depression severity was the primary outcome; all process variables were treated as secondary/exploratory outcomes for the purpose of examining proposed process variables.

#### 2.2.1 Primary outcome: depression

Depression severity was assessed using the Weinberger Adjustment Inventory [WAI (33);], an 84-item self-report scale measuring social and emotional adjustment. The WAI comprises 10 subscales grouped into three dimensions: Distress (Anxiety, Depression, Low Self-Esteem, Low Well-Being), Restraint (Suppression of Aggression, Impulse Control, Consideration of Others, Responsibility), and Defensiveness (Repressive Defensiveness, Denial of Distress). Items are scored on a 5-point Likert scale ranging from 1 (*False/Almost Never*) to 5 (*True/Always*). The WAI has demonstrated discriminant, concurrent, and predictive validity across diverse samples (33). The Depression subscale (7 items) served as the primary outcome, with total scores ranging from 7 to 35 and higher scores indicating greater depression severity. The WAI Depression subscale has demonstrated good internal consistency (*α* = .74-.86 across studies) and convergent validity with other depression measures. In the present sample, internal consistency was adequate at baseline (Cronbach’s *α* = .79).

#### 2.2.2 Process measures

All process variables described below were obtained from the publicly available dataset (32) as provided by the original authors; no additional composites were computed for this reanalysis. Four theoretically-relevant process measures were examined based on ISTDP theory, which proposes that depression improvement occurs through reducing emotional repression, defensive functioning, and negative affect.

##### 2.2.2.1 Emotional repression

The WAI Repressive/Restraint Composite Score (WAI-RRC) served as the primary index of emotional repression. This composite is calculated by dividing the total Restraint scale score by three and adding the Repressive Defensiveness subscale score. The Restraint scale (30 items) assesses suppression of emotional expression and impulse inhibition across four subscales (Suppression of Aggression, Impulse Control, Consideration of Others, Responsibility), while the Repressive Defensiveness subscale (11 items) measures denial and minimization of psychological distress. Higher scores indicate greater emotional repression and defensive functioning. This composite has shown sensitivity to change in psychodynamic psychotherapy research. The WAI-RRC was provided pre-computed in the dataset (32). Because it is a computed composite rather than a psychometric scale, internal consistency (Cronbach’s *α*) is not an appropriate reliability index for this measure.

##### 2.2.2.2 Negative affect

The Negative Affect subscale of the Positive and Negative Affect Schedule [PANAS (34);] assessed the intensity of negative emotional states. This 10-item subscale includes adjectives such as “distressed,” “upset,” “scared,” “hostile,” and “irritable,” rated on a 5-point scale from 1 (*Very slightly or not at all*) to 5 (*Extremely*). Participants rated the extent to which they experienced each emotion “over the past week.” Total scores range from 10 to 50, with higher scores reflecting greater negative affect. The PANAS Negative Affect subscale has demonstrated excellent psychometric properties across cultures (*α* typically *>*.85) and showed strong internal consistency in this sample (*α* = .89 at baseline).

##### 2.2.2.3 Overall distress

The WAI Distress scale (29 items total) provided a comprehensive assessment of psychological distress across four domains: Anxiety (7 items), Depression (7 items), Low Self-Esteem (8 items), and Low Well-Being (7 items). Importantly, this composite includes the Depression subscale, creating conceptual overlap when Depression serves as the outcome variable in mediation analyses (this caveat is discussed further in the Discussion section). Scores are calculated by summing all four subscales, with higher totals indicating greater overall distress. The WAI Distress scale has shown good convergent validity with other measures of psychopathology (*α* = .91 at baseline in this sample).

##### 2.2.2.4 Suppression of aggression

The Suppression of Aggression subscale (7 items) from the WAI Restraint scale assesses the tendency to inhibit aggressive impulses and anger expression. Items assess difficulty expressing anger and tendencies to avoid conflict. Higher scores indicate greater suppression of aggressive feelings and impulses (*α* = .76 at baseline).

#### 2.2.3 Measure selection rationale

These measures were selected based on ISTDP theory, which proposes specific processes of change. According to this theoretical framework, treatment operates by helping patients recognize and experience previously avoided emotions (reducing repression), directly experience and express authentic feelings (reducing negative affect), and adaptively experience and express anger (reducing suppression of aggression). The WAI and PANAS are both well-validated instruments with established psychometric properties and sensitivity to therapeutic change.

### 2.3 Procedure

#### 2.3.1 Treatment: ISTDP

Participants randomized to the active treatment condition received 20 individual ISTDP sessions delivered over 10 weeks (two 50-minute sessions per week). Treatment was delivered by two licensed clinical psychologists with specialized training in ISTDP who had completed formal certification programs and received ongoing supervision.

ISTDP is a brief psychodynamic psychotherapy developed by Davanloo (10) that aims to help patients access and experience previously avoided emotions, particularly grief, anger, and guilt (11). The therapist actively identifies and challenges defensive patterns that prevent emotional experiencing, using a collaborative therapeutic alliance to confront resistance to change. Through systematic attention to moment-to-moment shifts in affect, anxiety, and defensive functioning, the therapist helps patients break through defensive barriers to directly experience core emotions, which is theorized to produce lasting symptom relief (11, 14). Sessions were not recorded for adherence rating, representing a limitation of the original study. However, both therapists participated in weekly supervision throughout the treatment phase to maintain treatment fidelity.

#### 2.3.2 Control condition

Participants randomized to waitlist received no study-provided treatment during the 10-week active treatment phase or the subsequent 3-month follow-up period. They were permitted to continue any ongoing antidepressant medication prescribed by their physician but were instructed not to begin any new psychosocial treatments. Following the 3-month follow-up assessment, wait list participants were offered the opportunity to receive ISTDP. The use of an untreated waitlist control condition was ethically justified given that participants had previously failed to respond to pharmacotherapy and no evidence-based psychotherapy services were readily available in the study setting during the enrollment period.

#### 2.3.3 Assessment schedule

Outcome measures were administered at three time points:

1. *Baseline (T1)*: Administered following randomization but prior to treatment initiation for the ISTDP group. All participants completed assessments during the same week.
2. *Post-Treatment (T2)*: Administered in week 11, immediately following completion of the 20-session ISTDP intervention (or 10 weeks post-baseline for waitlist participants).
3. *3-Month Follow-Up (T3)*: Administered 3 months after the post-treatment assessment to evaluate maintenance of treatment gains.

Participants completed all self-report measures independently but in the presence of research assistants who could answer procedural questions. Assessments were conducted in quiet, private rooms. Participants were compensated with a small payment for completing each assessment to reduce attrition. To minimize expectancy effects inherent in waitlist designs, all participants received standardized information about the study procedures and were informed that waitlist participants would be offered treatment after the follow-up assessment; waitlist participants had no contact with study therapists during the waiting period.

### 2.4 Data analysis

All analyses were specified *a priori* (not pre-registered) and conducted on the complete dataset without interim analyses. The trajectory analyses for treatment effectiveness were confirmatory in nature; the mediation and cross-lagged analyses examining proposed process variables should be considered exploratory given the moderate sample size and limited number of assessment occasions. Statistical significance was evaluated at *α* = .05 (two-tailed) unless otherwise specified. All confidence intervals are reported at the 95% level.

#### 2.4.1 Trajectory analyses

Linear mixed-effects models (LMMs) were used to analyze trajectories of depression and process measures over time, providing several advantages over traditional repeated-measures ANOVA: (a) accommodation of missing data under the missing at random (MAR) assumption without listwise deletion, (b) modeling of individual-level heterogeneity through random effects, and (c) flexible handling of time structures.

For each outcome (depression and four process measures), we fit a model with the following specification:


$$Y_{ij}=\beta_{0}+\beta_{1}Time_{ij}+\beta_{2}Treatment_{i}+\beta_{3}{\left(Time\times Treatment\right)}_{ij}+u_{0i}+\in_{ij}$$

where *Y_ij_* represents the outcome for person *i* at time *j*, Time is a categorical factor (baseline, post-treatment, follow-up), Treatment is a binary indicator (ISTDP vs. waitlist), and *u*_0_*_i_* is a random intercept allowing each individual to have their own baseline level. Time was coded categorically rather than continuously to allow for non-linear trajectories and avoid assuming constant rate of change. Treatment was coded as 0 (waitlist) and 1 (ISTDP). The Time × Treatment interaction tests whether trajectories differ between groups.

Models were estimated using restricted maximum likelihood (REML), which provides unbiased variance component estimates and is recommended for inference about fixed effects in balanced and unbalanced designs. Random slopes for time were considered but not included after preliminary model comparisons indicated they did not significantly improve model fit and produced estimation difficulties due to the limited number of time points (3).

Significance of fixed effects was evaluated using Type III analysis of variance with Satterthwaite degrees of freedom. Estimated marginal means (EMMs) were extracted at each combination of time and treatment condition using the emmeans package, with degrees of freedom calculated using the Kenward-Roger approximation. Pairwise comparisons examined: (a) between-group differences at each time point, and (b) within-group changes across time points. No adjustments were made for multiple comparisons given the hierarchical nature of the hypotheses (primary interest in Time × Treatment interaction, with pairwise comparisons serving as follow-up tests).

Between-group effect sizes (Cohen’s *d*) were calculated at each time point using pooled standard deviations from observed data at that timepoint. This approach differs from the mixed-model-based effect size computation used in the original publication (24), which incorporated variance components across all timepoints. Consequently, our reported effect sizes may differ slightly from those in the original article, though both approaches provide valid estimates of treatment magnitude. Effect sizes were interpreted using conventional benchmarks: *d* = 0.20 (small), 0.50 (medium), 0.80 (large), with values *>* 1.20 considered very large.

#### 2.4.2 Model assumptions

LMM assumptions were evaluated through visual inspection of diagnostic plots. Normality of residuals was assessed via Q-Q plots and histograms. Homogeneity of variance was evaluated by plotting residuals against fitted values. Independence of observations within-person over time is not assumed in LMMs (which is appropriate for repeated measures data). No severe violations were detected for any model.

#### 2.4.3 Concurrent associations

To examine whether process changes were associated with depression changes, we calculated Pearson correlations between change scores (follow-up minus baseline) for each process measure and depression. These correlations were computed within the ISTDP group only (*n* = 43), as changes in the waitlist group were minimal. Change score correlations provide an index of whether individuals who showed greater improvement in process measures also showed greater depression improvement, though they do not establish temporal precedence or causality.

#### 2.4.4 Mediation analyses

We tested whether treatment effects on depression were mediated by changes in process measures using the causal mediation framework. This framework estimates the indirect effect of treatment on outcome that flows through the mediator, separating it from the direct effect. Identification of the average causal mediation effect relies on the sequential ignorability assumption—that no unmeasured confounders affect both the mediator and the outcome, conditional on treatment assignment and covariates. This assumption cannot be empirically verified and should be borne in mind when interpreting results.

Mediation and cross-lagged analyses were conducted for Emotional Repression (WAI-RRC), Negative Affect (PANAS), and Distress. Suppression of Aggression was not tested as a separate mediator because it is a component subscale of the WAI-RRC composite already included in the analyses.

For each process measure, we specified: - *Treatment (X)*: ISTDP vs. waitlist (binary indicator) – *Mediator (M)*: Change in process measure from baseline to post-treatment - *Outcome (Y)*: Depression at 3-month follow-up - *Covariate (C)*: Depression at baseline

The mediation model consists of two regressions:

Mediator model:


$$M_{i}=\alpha_{0}+\alpha_{1}X_{i}+\in_{Mi}$$

Outcome model:


$$Y_{i}=\beta_{0}+\beta_{1}M_{i}+\beta_{2}X_{i}+\beta_{3}C_{i}+\in_{Yi}$$

The average causal mediation effect (ACME; indirect effect) represents the expected change in follow-up depression due to treatment-induced changes in the mediator. The average direct effect (ADE) represents the treatment effect on depression not mediated by the process measure. The total effect is the sum of indirect and direct effects. The proportion mediated is calculated as ACME/(ACME + ADE).

Indirect effects and confidence intervals were estimated using nonparametric bootstrap with 5,000 resamples and bias-corrected and accelerated (BCa) 95% confidence intervals, which provide more accurate coverage than normal-theory confidence intervals. Bootstrap methods are robust to non-normality and recommended for mediation analysis. Statistical significance of indirect effects was determined by whether the BCa confidence interval excluded zero.

Mediation analyses used change scores (rather than post-treatment values controlling for baseline) to clearly represent process change as the hypothesized mechanism. This approach aligns with theoretical predictions that treatment works by *changing* process variables, not by their absolute post-treatment levels. A sensitivity analysis additionally controlling for baseline mediator values yielded virtually identical results, indicating that the findings are robust to potential regression-to-the-mean effects.

#### 2.4.5 Temporal precedence

Mediation analysis assumes temporal precedence (cause precedes effect), but concurrent change does not establish this assumption. We therefore conducted cross-lagged panel analyses to test whether: (a) earlier process levels predict later depression (process → depression), or (b) earlier depression levels predict later process measures (depression → process).

For each process measure, we tested both directional paths using ordinary least squares (OLS) regression examining the early period (baseline to post-treatment):


$$\text{Process}\to \text{Depression: }Depression_{T2,i}=\beta_{0}+\beta_{1}Process_{T1,i}+\beta_{2}Depression_{T1,i}+\beta_{3}Treatment_{i}+\in_{i}$$

$$\text{Depression}\to \text{Process: }Process_{T2,i}=\gamma_{0}+\gamma_{1}Depression_{T1,i}+\gamma_{2}Process_{T1,i}+\gamma_{3}Treatment_{i}+\in_{i}$$

The coefficients *β*_1_ and *γ*_1_ test temporal precedence, controlling for prior levels of the outcome (autoregressive effect) and treatment condition. Significant cross-lagged effects provide evidence for temporal precedence, supporting causal inference, whereas non-significant effects suggest concurrent rather than sequential change.

#### 2.4.6 Missing data

Of 258 total possible observations (86 participants × 3 time points), 22 observations (8.5%) had missing outcome data due to participant dropout (ISTDP: 7 dropouts [16.3%]; waitlist: 4 dropouts [9.3%]). All outcome variables showed the same missing data pattern, as missingness was due to complete dropout rather than item-level nonresponse. Little’s MCAR test suggested data were not missing completely at random, *χ*^2^*(45*) = 72.34, *p* = .006, indicating that missingness may be related to observed variables. Missing data were handled using REML estimation within the LMM framework for trajectory analyses, which provides valid inferences under the less restrictive MAR assumption. Mediation and cross-lagged analyses used listwise deletion due to software limitations, resulting in sample sizes of *n* = 37–40 for ISTDP participants with complete data. Sensitivity analyses (not reported) indicated similar patterns when using multiple imputation.

#### 2.4.7 Statistical power

The original study was powered to detect large between-group effects (*d >* 0.80) on depression with 80% power at *α* = .05. A sensitivity power analysis indicates that with *n* = 40 completers available for mediation analyses, the study had approximately 80% power to detect medium-sized indirect effects (ab = 0.39 standardized) at *α* = .05. Smaller indirect effects may therefore have gone undetected.

#### 2.4.8 Software

All analyses were conducted in R version 4.5.0 (35) using the following packages: *haven* (version 2.5.4) for importing SPSS data files; *lme4* (version 1.1-35.5) (36) and *lmerTest* (version 3.1-3) (37) for mixed-effects models; *emmeans* (version 1.10.0) (38) for estimated marginal means and pairwise contrasts; *effsize* (version 0.8.1) (39) for effect size calculations; *mediation* (version 4.5.0) (40) for bootstrap mediation analyses; and *papaja* (version 0.1.2) (41) for manuscript preparation in APA format. Complete analysis code is available on GitHub (https://github.com/robert-johansson/heshmati-reanalysis) and archived on Zenodo (https://doi.org/10.5281/zenodo.18743450).

## 3 Results

### 3.1 Participant characteristics

**Table 1** presents baseline demographic and clinical characteristics by treatment condition. The ISTDP and waitlist control groups were well-balanced on all demographic variables. The sample had a mean age of 36.90 years (*SD* = 11.73), with 61.6% female participants. Most participants were currently receiving antidepressant medication (79.1%) and had previously failed a mean of 1.8 antidepressant trials (*SD* = 0.9).

**Table 1:** Baseline demographic and clinical characteristics by treatment condition.

Characteristic	ISTDP (*n* = 43)	Waitlist control (*n* = 43)
Age, M (SD)	36.5 (12.3)	37.3 (11.3)
Gender, %		
Male	34.9	41.9
Female	65.1	58.1
Marital status, %		
Single	39.5	25.6
Married	51.2	60.5
Widowed/Divorced	9.3	14.0
Education, %		
High school	41.9	34.9
Undergraduate	37.2	48.8
Graduate	20.9	16.3
Employment status, %		
Employed	41.9	65.1
Unemployed	46.5	23.3
Retired	11.6	11.6
Socioeconomic status, %		
Low	14.0	16.3
Middle	65.1	65.1
High	20.9	18.6
Previous antidepressant trials, M (SD)	1.81 (1.01)	1.86 (0.89)
Currently receiving medication, %		
Yes	76.7	81.4
No	23.3	18.6

ISTDP, Intensive Short-Term Dynamic Psychotherapy.

### 3.2 Missing data

As described in the Methods section, 22 of 258 observations (8.5%) had missing data due to dropout, with identical patterns across all outcome variables. All analyses used the full intent-to-treat sample with REML estimation.

### 3.3 Primary outcome: depression trajectories

**Figure 1:**
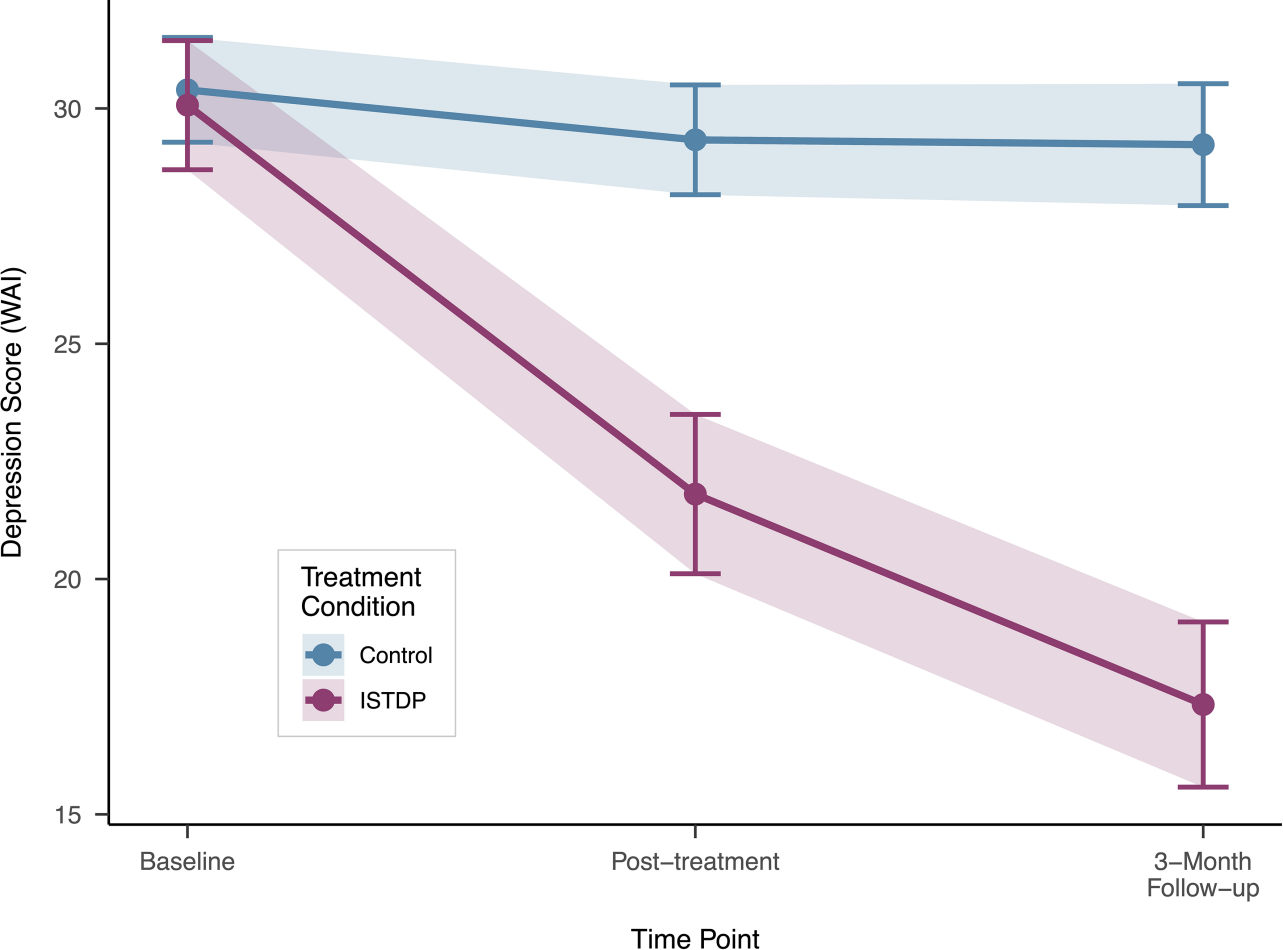
Mean depression trajectories by treatment condition. Error bars represent 95% confidence intervals. ISTDP, Intensive Short-Term Dynamic Psychotherapy.

**Figure 1** displays the depression trajectories for both treatment groups across the three assessment points. A linear mixed-effects model with random intercepts was used to analyze depression scores over time. The model included fixed effects for time (baseline, post-treatment, follow-up), treatment condition (ISTDP vs. waitlist control), and their interaction.

The overall Time × Treatment interaction was highly significant, *F*(2, 151.9) = 94.85, *p<*.001, indicating that the two groups showed significantly different trajectories of change over time. Specifically, the interaction was significant at both post-treatment, $\hat{\beta }=-7.29$, 95% CI [−8.97,−5.61], *t*(153.73) = −8.51, 426 *p<.*001, and follow-up, $\hat{\beta }=-11.66$, 95% CI [−13.34,−9.98], *t*(153.73) = −13.61, *p<.*001.

#### 3.3.1 Estimated marginal means

**Table 2** presents the estimated marginal means for depression scores at each time point by treatment condition. At baseline, the groups did not differ significantly in depression levels, *t*(133.0) = 0.34, *p* = .734, Cohen’s *d* = 0.08, 95% CI [-0.35, 0.51].

**Table 2:** Estimated marginal means for depression scores by treatment condition and time point.

Time point	ISTDP			Waitlist control		
	*M*	*SE*	95% CI	*M*	*SE*	95% CI
Baseline	30.07	0.68	[28.73, 31.41]	30.40	0.68	[29.06, 31.73]
Post-treatment	21.67	0.71	[20.26, 23.08]	29.29	0.70	[27.92, 30.67]
3-Month Follow-up	17.20	0.71	[15.79, 18.61]	29.19	0.70	[27.81, 30.56]

Values are estimated marginal means from the linear mixed-effects model with random intercepts. CI, 95% confidence interval; ISTDP, Intensive Short-Term Dynamic Psychotherapy.

However, at post-treatment, ISTDP participants showed significantly lower depression scores than waitlist controls, *t*(146.7) = 7.65, *p<*.001, Cohen’s *d* = 1.68, 95% CI [1.15, 2.22], representing a large effect. This between-group difference increased further at 3-month follow-up, *t*(146.7) = 12.03, *p<*.001, Cohen’s *d* = 2.50, 95% CI [1.88, 3.11], representing a very large effect.

#### 3.3.2 Within-group changes

For ISTDP participants, depression scores decreased significantly from baseline to post-treatment (*M*_diff_= 8.40, *SE* = 0.62, *p<*.001) and continued to decrease from baseline to 3-month follow-up (*M*_diff_ = 12.87, *SE* = 0.62, *p<*.001). Additional improvement occurred between post-treatment and follow-up (*M*_diff_ = 4.47, *SE* = 0.63, *p<*.001).

In contrast, waitlist control participants showed minimal change in depression scores across all time points (all *p*s *>*.10), with no significant differences between baseline and post-treatment (*M*_diff_ = 1.10, *p* = .156), baseline and follow-up (*M*_diff_ = 1.21, *p* = .110), or post-treatment and follow-up (*M*_diff_ = 0.10, *p* = .984).

### 3.4 Model diagnostics

The random intercept variance was 12.59, indicating substantial between-person variability in baseline depression levels. The residual variance was 7.04. Visual inspection of residual plots (not shown) indicated acceptable model fit, with approximately normally distributed residuals and homogeneous variance across predicted values.

Having established that ISTDP produces large, sustained reductions in depression, we next examined whether these improvements operate through changes in theoretically-relevant process measures, including emotional repression, defensiveness, and negative affect—key targets of ISTDP theory.

### 3.5 Process measure changes

ISTDP theory proposes that depression improvement occurs through reducing emotional repression and defensiveness. We examined four theoretically-relevant process measures: Emotional Repression (WAI Repressive/Restraint Composite), Negative Affect (PANAS), overall Distress, and Suppression of Aggression. Linear mixed-effects models with random intercepts were fit for each process measure using the same analytical approach as the primary depression outcome.

**Figure 2:**
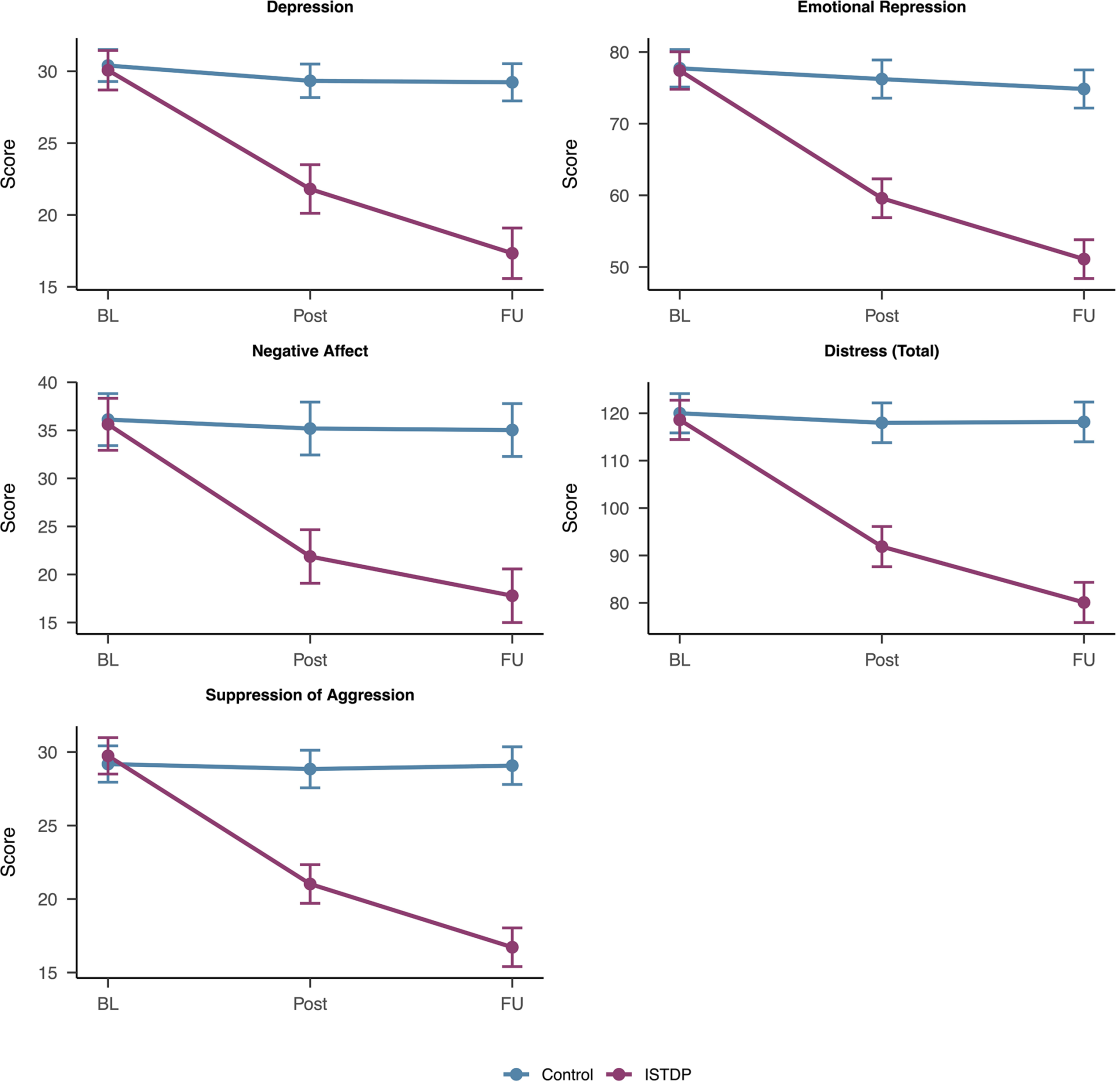
Trajectories for depression and key process measures by treatment condition. Error bars represent 95% confidence intervals. ISTDP, Intensive Short-Term Dynamic Psychotherapy; WAI-RRC, Weinberger Adjustment Inventory Repressive/Restraint Composite; PANAS, Positive and Negative Affect Schedule.

All four process measures showed highly significant Time × Treatment interactions (all *p*s<.001), indicating that ISTDP produced differential changes compared to waitlist control. **Figure 2** displays the trajectories for these key process measures alongside depression.

Effect sizes for process measures at 3-month follow-up were comparable to or exceeded the depression effect. Emotional Repression showed a very large effect (*d* = 2.76), as did Negative Affect (*d* = 1.96), Distress (*d* = 2.95), and Suppression of Aggression (*d* = 2.75).

Within the ISTDP group, baseline-to-follow-up changes in these process measures showed varying associations with depression change. Distress change was strongly correlated with depression change (*r* = 0.70), though this is expected given that the Distress composite includes the Depression subscale. In contrast, Negative Affect change (*r* = -0.19) and Emotional Repression change (*r* = 0.03) showed weak associations with depression change, suggesting that individual-level variation in these process changes does not track closely with depression improvement despite the large group-level treatment effects.

### 3.6 Mechanisms: mediation analyses

While ISTDP clearly affected hypothesized process measures, the critical question is whether these changes *mediate* (explain) depression improvement. We tested whether changes in process measures from baseline to post-treatment predicted depression at 3-month follow-up (controlling for baseline depression), using bootstrap mediation analysis with 5,000 resamples (**Table 3**).

**Table 3:** Mediation analysis results for key process measures.

Mediator	Indirect effect [95% CI]	Proportion mediated	p
Distress (Total)	-6.34 [-11.07, -3.08]	53.9%	0.000
Emotional Repression	-1.84 [-5.41, 1.00]	15.6%	0.246
Negative Affect	1.51 [-0.52, 3.87]	-12.8%	0.149

Indirect effects estimated using bootstrap mediation analysis with 5,000 resamples. CI = 95% bias-corrected and accelerated confidence interval. All models control for baseline depression. Negative indirect effects indicate mediation in the expected direction (treatment reduces mediator, which reduces depression). A positive indirect effect (as for Negative Affect) indicates the indirect path operates counter to the total treatment effect, resulting in a negative proportion mediated.

#### 3.6.1 Distress

Changes in overall distress significantly mediated depression improvement, with an indirect effect of -6.34, 95% CI [-11.07, -3.08], *p* =<.001, accounting for 53.9% of the total treatment effect. However, this finding must be interpreted cautiously as the Distress composite includes the Depression subscale itself, creating conceptual overlap between mediator and outcome.

#### 3.6.2 Emotional repression

Contrary to theoretical expectations, changes in emotional repression did not significantly mediate depression improvement, indirect effect = -1.84, 95% CI [-5.41, 1.00], *p* = .246. This null finding is theoretically surprising, as reducing emotional repression is a core hypothesized mechanism in ISTDP.

#### 3.6.3 Negative affect

Changes in negative affect also did not significantly mediate depression improvement, indirect effect = 1.51, 95% CI [-0.52, 3.87], *p* = .149. Notably, the direction of this indirect effect was positive, meaning that treatment-induced reductions in negative affect were, if anything, associated with *less* depression improvement at follow-up—counter to the theoretical prediction. Although non-significant, this unexpected direction further suggests that negative affect reduction does not operate as a simple mediating pathway in ISTDP.

#### 3.6.4 Sensitivity analysis: distress without depression

To address the construct overlap between the Distress composite (which includes the Depression subscale) and the depression outcome, we conducted a sensitivity analysis removing the Depression subscale from the Distress composite. When mediation was tested using this reduced Distress measure (Anxiety + Low Self-Esteem + Low Well-Being), the indirect effect was non-significant, ACME = 0.55, 95% CI [-1.98, 3.27], *p* = .702. This confirms that the significant Distress mediation finding was driven by the shared Depression content, not by anxiety, self-esteem, or well-being changes independently mediating depression improvement.

### 3.7 Temporal precedence

The null mediation findings for the theoretically central process measures (emotional repression, negative affect) prompted examination of temporal dynamics. Cross-lagged analyses tested whether process measures at baseline predicted depression at post-treatment (controlling for baseline depression and treatment condition), and vice versa. All variables were standardized to yield standardized regression coefficients (*β*).

Results revealed no clear temporal precedence for any process measure. For Distress, neither direction showed significant cross-lagged effects (process → depression: *β* = -0.03, 95% CI [-0.29, 0.22], *p* = .808; depression → process: *β* = 0.03, 95% CI [-0.09, 0.15], *p* = .619). Similarly, Emotional Repression (process → depression: *β* = -0.11, 95% CI [-0.25, 0.02], *p* = .098) and Negative Affect (process → depression: *β* = 0.09, 95% CI [-0.05, 0.22], *p* = .217) showed no evidence that process changes preceded depression changes.

This pattern suggests that process measures and depression change *concurrently* rather than sequentially. Rather than a specific mechanism where process changes lead to depression improvement (Treatment → Process → Depression), ISTDP appears to create simultaneous change across multiple domains (Treatment → Process AND Depression).

## 4 Discussion

This reanalysis examined the effects and proposed process variables of Intensive Short-Term Dynamic Psychotherapy (ISTDP) for treatment-resistant depression using public data from a randomized controlled trial (*N* = 86). Four principal findings emerged. First, ISTDP produced large effects on depression at post-treatment (*d* = 1.68) that continued to increase at 3-month follow-up (*d* = 2.50), confirming and extending the original study findings. Second, ISTDP produced comparable large effects on all proposed process measures (emotional repression *d* = 2.76; negative affect *d* = 1.96; distress *d* = 2.95). Third, contrary to theoretical predictions, mediation analyses indicated that no conceptually independent process measure significantly mediated depression improvement—distress showed apparent mediation, but a sensitivity analysis removing its overlapping Depression subscale eliminated this effect. Fourth, cross-lagged analyses revealed no temporal precedence, suggesting concurrent rather than sequential change. These findings confirm ISTDP’s effectiveness for treatment-resistant depression while challenging current theoretical assumptions about how this treatment works.

### 4.1 Large and durable treatment effects on depression

The magnitude and pattern of ISTDP’s effects on depression in this study are very large relative to waitlist control. The between-group effect size at 3-month follow-up (*d* = 2.50, 95% CI [1.88, 3.11]) substantially exceeds typical benchmarks for psychotherapy outcomes. Meta-analytic evidence indicates that psychotherapy for depression generally produces effect sizes around *d* = 0.90 (7), while specialized interventions for treatment-resistant depression show more modest effects (*d* = 0.60 in the CoBalT trial (6). However, effect sizes relative to waitlist controls are typically inflated due to nocebo effects and expectancy factors (7). Comparisons with active treatment conditions (e.g., cognitive-behavioral therapy or other evidence-based interventions) would provide a more conservative estimate of ISTDP-specific effects. The present findings replicate previous ISTDP studies showing large effects for treatment-resistant populations (9, 22), with effect sizes comparable to or exceeding those reported in the original publication (24).

Particularly noteworthy is that effect sizes *increased* over time rather than diminishing after treatment ended. Depression improvements were large at post-treatment (*d* = 1.68) and grew substantially larger by 3-month follow-up (*d* = 2.50), with ISTDP participants showing continued symptom reduction between post-treatment and follow-up (*M*_diff_ = -4.47 points, *p<*.001). This pattern of continued improvement post-treatment is relatively uncommon in psychotherapy research and suggests that ISTDP may equip patients with skills, insights, or capacities that continue to benefit them after active treatment ends. This is consistent with the theoretical model underlying ISTDP, which proposes that accessing and processing previously avoided emotions produces fundamental shifts in psychological functioning rather than temporary symptom suppression (11, 12). Similar patterns of sustained or increasing effects have been observed in other process oriented therapies such as cognitive therapy (42) and compassion-focused therapy (43), suggesting that treatments targeting underlying psychological processes may produce more durable benefits than those focused solely on symptom management.

The clinical significance of these effects is substantial. ISTDP participants experienced an average reduction of 12.87 points on the 7–35 point WAI Depression subscale from baseline to follow-up, representing a 45% reduction in depression scores (baseline *M* = 28.81 to follow-up *M* = 15.94). In contrast, waitlist control participants showed essentially no change across the same period (total change = -0.21 points,<1% reduction). This large within-person change in the ISTDP group indicates clinically meaningful symptom reduction. The stability of waitlist control scores rules out alternative explanations such as spontaneous remission, regression to the mean, or time-related effects, strengthening the causal inference that observed improvements resulted from ISTDP treatment itself.

Our reanalysis confirmed the large treatment effects reported in the original study, consistent with a growing body of evidence supporting ISTDP as an effective intervention for depression (8, 44), including treatment-resistant presentations (9, 22).

### 4.2 Substantial changes in proposed process measures

ISTDP not only produced large effects on depression but also showed very large effects on all hypothesized process measures. Effect sizes at 3-month follow-up reached *d* = 2.76 for emotional repression, *d* = 1.96 for negative affect, *d* = 2.95 for overall distress, and *d* = 2.75 for suppression of aggression. These magnitudes equal or exceed the depression effect itself, indicating that ISTDP engaged its hypothesized therapeutic targets and produced broad psychological change beyond symptom reduction alone.

This pattern validates a core premise of ISTDP theory: that the treatment works by targeting defensive processes and emotional experiencing. Patients in the ISTDP condition showed substantial reductions in emotional repression (indicating less defensive avoidance of feelings), decreased negative affect (suggesting improved emotional regulation), and lower overall distress across multiple psychological domains. These changes demonstrate that ISTDP was doing what it was designed to do—helping patients access and process previously avoided emotions, reduce defensive functioning, and experience improved psychological well-being across multiple dimensions (11, 12).

However, this creates a puzzle: If ISTDP produces large group-level effects on both hypothesized process variables and depression, why don’t these process changes mediate depression improvement? Within the ISTDP group, only Distress change correlated substantially with depression change (*r* = 0.70)—but this correlation is inflated by shared item content. Emotional Repression (*r* = 0.03) and Negative Affect (*r* = -0.19) showed weak within-group associations with depression change. This pattern—large treatment effects on hypothesized mediators but weak individual-level associations with the outcome—represents a fundamental challenge to theoretical assumptions about how ISTDP works.

### 4.3 The mediation puzzle: interpretation and implications

#### 4.3.1 Exploratory mediation findings

Despite large treatment effects on both process measures and depression, emotional repression and negative affect did not significantly mediate depression improvement. Distress showed apparent mediation (54% of total effect), but this finding is attributable to the Depression subscale within the Distress composite: a sensitivity analysis removing this subscale eliminated the mediation effect entirely (ACME = 0.55, *p* = .702). Furthermore, within-group correlations between process change and depression change were weak for the conceptually distinct measures (Emotional Repression *r* = 0.03; Negative Affect *r* = -0.19), indicating that individual variation in process change does not track with depression improvement.

In these exploratory analyses, the core process variables proposed by ISTDP theory—reducing emotional repression and facilitating emotional experiencing—did not demonstrate the expected mediating role. While these findings must be interpreted cautiously given the moderate sample size and limited measurement occasions, they suggest a need for theoretical refinement. ISTDP theory explicitly predicts a sequential pathway: treatment helps patients break through defensive barriers (reducing repression), which allows them to access and process previously avoided emotions (reducing negative affect), which in turn alleviates depressive symptoms (11–13). The present data suggest a different pattern: treatment produces simultaneous change across multiple domains rather than working through a specific sequential mechanism. However, an important caveat is that the self-report measures used here may not adequately capture the unconscious processes central to ISTDP theory; we return to this measurement issue below.

This finding is not unique to ISTDP. Kazdin (25) has documented that identifying mechanisms of therapeutic change remains one of the most challenging problems in psychotherapy research, with many established treatments showing unclear or inconsistent evidence for their proposed mechanisms despite robust evidence of effectiveness. Similarly, a recent meta-analysis of mediation studies across depression interventions found that while cognitive and behavioral change were commonly tested as mediators, the evidence for specific proposed mechanisms remained inconsistent (45). The present findings thus join a broader pattern in psychotherapy science: demonstrating that a treatment works is difficult; understanding *how* it works is even more challenging.

#### 4.3.2 Possible explanations for null mediation

##### 4.3.2.1 Measurement timing and temporal resolution

One plausible explanation is that three measurement occasions (baseline, post-treatment, 3-month follow-up) provide insufficient temporal resolution to capture the dynamic processes through which ISTDP operates. Process changes may occur within or between individual therapy sessions at a much finer temporal grain than our measurement intervals could detect. If emotional experiencing and defensive restructuring fluctuate session-by-session or even moment-tomoment within sessions (46), aggregating these processes into change scores across 10-week intervals may obscure the true temporal dynamics.

Moreover, research on sudden gains in psychotherapy suggests that therapeutic improvement often occurs in discrete, non-linear breakthroughs rather than through gradual, continuous change (47). Sudden gains have been documented in psychodynamic therapy at rates comparable to cognitive-behavioral therapy (48), and in supportive-expressive therapy, therapist interpretation accuracy—rather than cognitive change— predicted these gains (49). ISTDP, with its emphasis on in-session emotional breakthroughs and “unlocking the unconscious,” may be particularly prone to producing such discontinuous change. If therapeutic gains occur in critical moments during sessions, they would not be captured by assessments administered months apart.

This explanation receives support from research using more fine-grained measurement. Town et al. (29) examined session-by-session processes in an ISTDP trial for treatment-resistant depression, finding that in-session experiencing of anger during early sessions predicted subsequent depression reductions measured the following week. This suggests that therapeutic processes operate at a weekly or even daily timescale, not the months-long intervals available in the present data. Similarly, work using ecological momentary assessment in psychotherapy has revealed within-day fluctuations in affect and symptoms that predict therapeutic outcomes (50), dynamics that would be invisible in traditional pre-post designs.

However, temporal resolution cannot be the complete explanation. Even with only three timepoints, if process changes from baseline to post-treatment were causally driving depression changes from post-treatment to follow-up, we would expect to see this pattern in both the mediation analyses and the cross-lagged analyses. We found neither. This suggests that the issue extends beyond measurement timing alone.

##### 4.3.2.2 Measurement validity and the unconscious

A second explanation concerns whether self-report questionnaires can adequately capture the unconscious processes central to ISTDP theory. ISTDP posits that therapeutic change occurs through “unlocking the unconscious”—a process in which patients access and directly experience emotions that have been kept out of awareness through defensive processes (10, 11). By definition, these are processes that patients cannot consciously report on until they occur in therapy.

This creates a fundamental measurement paradox: Can patients accurately self-report on defensive processes designed to keep material out of awareness? The WAI Repressive/Restraint Composite, while psychometrically sound, assesses trait-like defensive styles through questions like “I keep my feelings under control” and “I rarely get angry.” Patients may report feeling less defensive over time, but this self-reported change may not capture the in-session experiential breakthroughs that ISTDP theory identifies as the active therapeutic process (13).

Critically, Johansson et al. (13) found that when “unlocking the unconscious” was measured through observer ratings of session videos—capturing actual in-session emotional breakthroughs—this process *did* significantly mediate depression outcomes with a moderate effect size (*d* = 0.60). Patients who experienced unlocking during treatment showed larger improvements (*d* = 1.32) than those who did not (*d* = 0.72). This suggests that the proposed process may be valid but requires observational measurement methods that can capture experiential processes as they unfold in therapy. Observer-rated instruments such as the Achievement of Therapeutic Objectives Scale (51) and observer-coded affective processes (46) offer more direct indices of the in-session changes that ISTDP theory identifies as central. Self-report measures administered at months-long intervals may simply be the wrong tool for assessing these processes.

##### 4.3.2.3 True concurrent change and theoretical revision

A third possibility is that ISTDP does not actually work through the proposed sequential pathway, and the theory requires revision. Perhaps emotional repression, negative affect, and depression do not have a causal relationship but rather change concurrently as part of a broader therapeutic process. Under this interpretation, ISTDP creates a therapeutic context characterized by strong alliance, active engagement, emotional activation, and therapist responsiveness (52). Within this context, multiple psychological domains improve simultaneously—patients feel less defensive, experience less negative emotion, and feel less depressed—but not in a specific causal sequence.

##### 4.3.2.4 The cross-lagged analyses support this interpretation

We found no evidence that earlier process levels predicted later depression changes, nor that earlier depression predicted later process changes. Both process measures and depression changed together over the same timeframes, consistent with concurrent rather than sequential change. This pattern suggests that ISTDP may operate through what Wampold and Imel (52) describe as common factors—alliance quality, therapist empathy, patient engagement, and hope—rather than through technique-specific processes alone. In ISTDP, the therapist-patient relationship is itself a central vehicle for change: the therapist actively engages the patient in a collaborative effort to confront defensive avoidance, and it is within this relational context that emotional breakthroughs occur (11). Town et al. (29) demonstrated that the therapeutic alliance mediated the relationship between in-session anger experiencing and depression outcomes in ISTDP, suggesting that relational and technical factors are deeply intertwined rather than separable.

An alternative theoretical refinement is that ISTDP may alleviate depression not by eliminating repression entirely—through full “unlocking of the unconscious”—but by facilitating a shift in defensive organization. For instance, patients may move from repression (keeping emotions fully out of awareness) to isolation of affect (being cognitively aware of feelings but affectively disconnected), a less pathological and less energy intensive defensive style (11). Such defensive restructuring could reduce depression without requiring the complete mediational pathway that ISTDP theory traditionally proposes.

##### 4.3.2.5 This interpretation does not diminish ISTDP’s value

Many highly effective medical interventions worked for decades before their mechanisms were understood (aspirin being the classic example). However, it does challenge the field to refine its theoretical models. Rather than a linear pathway (Treatment → Reduce Repression → Reduce Depression), ISTDP may create synergistic change across multiple interacting systems, with depression improvement emerging from broad shifts in emotional processing, self-concept, relationship patterns, and defensive functioning occurring in concert.

##### 4.3.2.6 Individual differences and multiple pathways

A fourth explanation emphasizes heterogeneity across patients. ISTDP theory itself distinguishes between psychodiagnostic groups—resistant, repressive, and fragile—each characterized by distinct defensive organizations, anxiety discharge patterns, and corresponding treatment algorithms (11, 27). Mechanisms of change likely differ across these groups: for resistant patients, breaking through tactical defenses may be the critical process; for repressive patients, mobilizing warded-off affect; for fragile patients, building capacity to tolerate emotional experience. When these distinct pathways are averaged together in group-level mediation analyses, the result may be null findings despite meaningful processes operating within each subgroup.

##### 4.3.2.7 Evidence supports this possibility

Town et al. (29) found that personality pathology moderated process pathways: For patients with lower personality pathology, in-session anger experiencing predicted outcomes, whereas for patients with higher pathology, this same process required strong therapeutic alliance to be beneficial. This suggests that who benefits from what process depends on patient characteristics. Modern precision medicine approaches in psychotherapy emphasize such moderation effects (53, 54), recognizing that “one size fits all” models fail to capture the complexity of therapeutic change.

Testing this explanation requires person-specific analytic approaches (55) or adequately powered moderation analyses to identify for whom different therapeutic processes operate. The present sample (*N* = 86) lacks statistical power to detect moderation effects reliably, but future research with larger samples and planned moderation analyses could illuminate whether different patients improve through different pathways.

##### 4.3.2.8 The distress confound

The one apparently significant mediation finding—that distress mediated 54% of depression improvement—is attributable to shared item content. Our sensitivity analysis removing the Depression subscale from the Distress composite eliminated the mediation effect (ACME = 0.55, *p* = .702), confirming that this finding reflected construct overlap rather than a genuine mediational pathway. This leaves us with the conclusion that neither emotional repression nor negative affect, the two core process variables proposed by ISTDP theory that are conceptually distinct from the outcome, demonstrated significant mediation.

Taken together, the present findings establish that ISTDP produces very large, durable improvements in depression for treatment-resistant patients and large concurrent changes in proposed process measures. What remains unclear is whether these process changes *cause* depression improvement or simply co-occur, and what the active processes of change actually are. The evidence is most consistent with a model of broad, concurrent therapeutic change rather than a specific linear causal pathway, though this interpretation must be considered tentative. With only three measurement occasions spanning months-long intervals, we cannot capture the fine-grained temporal dynamics through which therapeutic processes may operate, and self-report measures at discrete assessment points may not adequately capture the unconscious emotional processes that ISTDP theory identifies as central to change (13). Critically, however, the unclear process evidence does not challenge the effectiveness evidence. Understanding precisely how ISTDP works requires multi-method assessment approaches capturing both self-reported and observer-rated processes, and intensive longitudinal designs with finer temporal resolution.

### 4.4 Clinical implications

Despite the large effects observed, the limited number of ISTDP trials warrants caution in clinical recommendations. The concurrent change pattern suggests that the therapeutic benefits may not depend on strict adherence to a specific technical sequence, and that common therapeutic factors (52) may play an important role alongside ISTDP-specific techniques. Future research with larger samples and more diverse populations is needed before firm clinical guidelines can be established.

### 4.5 Limitations

Several limitations qualify these findings and warrant consideration. First, as a secondary analysis, this study was constrained by the original design decisions, including measurement selection, assessment timing, and sample characteristics. We had no input into which process measures were included, limiting our ability to test alternative mediators that might better capture ISTDP therapeutic processes. Most critically, only three measurement occasions—while sufficient for testing treatment effectiveness—provide limited temporal resolution for investigating dynamic therapeutic processes. Mechanisms may unfold session-by-session or even moment-to-moment, dynamics invisible to assessments conducted months apart.

Second, reliance exclusively on self-report measures introduces limitations. Self-report cannot capture unconscious processes by definition, creating a fundamental mismatch between measurement method and theoretical constructs. The contrast with Johansson et al. (13), who found significant mediation using observer-rated measures of unlocking, suggests that observational coding of therapy sessions might reveal processes invisible to questionnaires. Similarly, behavioral tasks, physiological measures, or clinician ratings might provide complementary perspectives on therapeutic processes.

Third, the measure of distress included the depression outcome as a component, rendering the one significant mediation finding uninterpretable due to circularity. Future process research should ensure conceptual and operational independence between proposed mediators and outcomes.

Fourth, the waitlist control design, while ethically justified given the treatment-resistant population and limited access to psychotherapy in the study setting, precludes conclusions about ISTDP-specific processes versus general psychotherapy factors. Comparisons with other active treatments (e.g., cognitive-behavioral therapy, interpersonal psychotherapy) would clarify whether observed effects and processes are specific to ISTDP or shared across therapeutic approaches.

Fifth, the sample comprised Iranian adults, and cultural factors may influence both depression presentation and therapeutic processes. Generalizability to other cultural contexts requires empirical verification. Additionally, focusing exclusively on treatment-resistant depression limits generalizability to first-episode or less severe presentations, though these patients clearly represent the population for whom alternative interventions are most urgently needed.

Sixth, the moderate sample size (*N* = 86) provided adequate power for detecting large main effects but limited power for mediation analyses seeking to detect small-to-moderate indirect effects and inadequate power for testing moderation hypotheses. Additionally, approximately 13% missing data due to dropout, though handled appropriately through REML estimation, may have introduced bias despite sophisticated missing data methods.

Seventh, treatment was delivered by two therapists, and therapist effects could influence outcomes. A sensitivity analysis adding therapist as a fixed effect showed no significant therapist main effect on depression (*χ*^2^ = 0.18, *p* = .672) and no Time × Therapist interaction within the ISTDP group (*F*(2, 72.6) = 0.23, *p* = .795), with estimated marginal means essentially unchanged. Results for process measures were similarly robust, though one therapist’s patients showed somewhat different negative affect trajectories (*p* = .034). Given only two therapists, these results should be interpreted cautiously, and future research with more therapists should model therapist as a random effect.

Finally, mediation analysis rests on strong untestable assumptions, most critically that no unmeasured confounders affect both mediator and outcome (sequential ignorability). Alternative causal structures could produce the observed patterns, and we cannot definitively rule out competing explanations. The cross-lagged analyses provide some additional evidence regarding temporal precedence, but even these cannot establish causation conclusively without experimental manipulation of proposed process variables.

### 4.6 Future directions

Several research directions could advance understanding of ISTDP therapeutic processes. Most importantly, studies with intensive temporal resolution—weekly or daily assessments throughout treatment— could capture therapeutic processes as they unfold (29). Ecological momentary assessment using smartphone-based diaries could track symptoms, affect, and defensive processes in real-world contexts multiple times per day, revealing within-person dynamics invisible to traditional assessment schedules (50). Such intensive longitudinal designs would enable dynamic systems modeling approaches that can represent the complex, reciprocal relationships among therapeutic processes.

Multi-method assessment batteries combining self-report, observational coding of therapy sessions, therapist ratings, behavioral tasks, and potentially physiological measures would provide triangulated evidence less susceptible to method-specific limitations. Observational coding of “unlocking the unconscious” (13), emotional experiencing (29), affective processes (46), and defensive functioning using validated instruments such as the Achievement of Therapeutic Objectives Scale (51) could capture processes that self-report questionnaires miss. Physiological markers such as heart rate variability, skin conductance, or cortisol might provide objective indices of emotional arousal and regulation relevant to hypothesized therapeutic processes.

Comparative effectiveness trials comparing ISTDP with other evidence-based treatments (cognitive-behavioral therapy, interpersonal psychotherapy, medication optimization) would clarify whether ISTDP’s large effects are specific to this approach or shared across effective treatments. Component analyses dismantling ISTDP into constituent elements (e.g., alliance-building, defense identification, emotional experiencing facilitation) could identify which components are necessary versus merely helpful, potentially allowing treatment streamlining and efficiency gains.

Adequately powered moderation analyses could identify for whom ISTDP works best and through which pathways. Potential moderators include baseline depression severity, personality pathology (suggested by Town et al. (29)), attachment style, emotion regulation capacity, and treatment history. Person-specific analytic approaches (55) could test whether different individuals improve through different pathways, with aggregated analyses obscuring this heterogeneity.

Extended follow-up assessments at 6, 12, and 24 months would establish long-term durability and identify factors predicting maintenance versus relapse. Understanding the processes underlying sustained improvement—how initial therapeutic experiences translate into lasting change—is critical for developing relapse prevention strategies and optimizing long-term outcomes.

Finally, independent replication in diverse samples, settings, and cultural contexts by research teams without allegiance to ISTDP would provide the strongest evidence for robustness and generalizability. The open availability of data and reproducible analysis code from this and the original study (24, 32) facilitates such replication efforts, exemplifying how open science practices advance cumulative knowledge.

### 4.7 Conclusions

These findings underscore a fundamental challenge in psychotherapy research: demonstrating that a treatment works is difficult; understanding how it works is even more challenging. ISTDP clearly produces very large benefits for patients with treatment-resistant depression—among the largest effects observed in the psychotherapy literature—with improvements that are durable and increase after treatment ends. These effectiveness findings position ISTDP as a valuable treatment option for a challenging clinical population facing limited alternatives.

However, the processes underlying these improvements remain to be fully elucidated. The fact that the present exploratory analyses did not identify the proposed sequential pathways—wherein reducing emotional repression leads to improved affect regulation which in turn alleviates depression—does not diminish ISTDP’s therapeutic value, but rather highlights the complexity of therapeutic change and the limitations of current methodological approaches to studying it. Several explanations merit consideration: insufficient temporal resolution of measurement, inability of self-report instruments to capture unconscious processes, true concurrent rather than sequential change, individual heterogeneity in process pathways, or some combination of these factors.

This disconnect between clear effectiveness evidence and unclear process evidence is not unique to ISTDP but reflects a broader pattern across psychotherapy research (25, 56). Many well-established treatments show robust efficacy despite inconsistent or weak evidence for their proposed processes. This creates both a practical reality and a research imperative. Practically, clinicians may consider ISTDP as a treatment option for patients with treatment-resistant depression, noting that the available evidence, while promising, comes primarily from waitlist-controlled trials and requires replication against active comparators. From a research perspective, the field needs refined theoretical models that can accommodate concurrent, multi-dimensional change; improved measurement approaches combining self-report, observational, and physiological methods; and intensive longitudinal designs capturing temporal dynamics at relevant timescales.

Future research with finer temporal resolution, multi-method assessment, attention to individual differences, and replication across diverse contexts will be essential to unraveling the pathways through which ISTDP—and psychotherapy more broadly—produces therapeutic change. Until then, the available evidence suggests that ISTDP is a promising treatment for treatment-resistant depression that warrants further investigation. Understanding therapeutic processes remains an important scientific goal, and the present findings highlight the need for refined measurement approaches to advance this understanding.

## Data Availability

The dataset analyzed in this study is publicly available on the Open Science Framework at https://doi.org/10.17605/OSF.IO/75PU8. Complete analysis code is archived on Zenodo (https://doi.org/10.5281/zenodo.18743450) and available at https://github.com/robert-johansson/heshmati-reanalysis.
